# The Gut-Brain Axis, the Human Gut Microbiota and Their Integration in the Development of Obesity

**DOI:** 10.3389/fphys.2018.00900

**Published:** 2018-07-12

**Authors:** Edward S. Bliss, Eliza Whiteside

**Affiliations:** School of Health and Wellbeing, University of Southern Queensland, Toowoomba, QLD, Australia

**Keywords:** gut-brain axis, microbiota, cholecystokinin (CCK), glucagon-like peptide 1 (GLP1), peptide YY_3−36_ (PYY), lipopolysaccharide (LPS), obesity, short-chain fatty-acids (SCFA)

## Abstract

Obesity is a global epidemic, placing socioeconomic strain on public healthcare systems, especially within the so-called Western countries, such as Australia, United States, United Kingdom, and Canada. Obesity results from an imbalance between energy intake and energy expenditure, where energy intake exceeds expenditure. Current non-invasive treatments lack efficacy in combating obesity, suggesting that obesity is a multi-faceted and more complex disease than previously thought. This has led to an increase in research exploring energy homeostasis and the discovery of a complex bidirectional communication axis referred to as the gut-brain axis. The gut-brain axis is comprised of various neurohumoral components that allow the gut and brain to communicate with each other. Communication occurs within the axis via local, paracrine and/or endocrine mechanisms involving a variety of gut-derived peptides produced from enteroendocrine cells (EECs), including glucagon-like peptide 1 (GLP1), cholecystokinin (CCK), peptide YY_3−36_ (PYY), pancreatic polypeptide (PP), and oxyntomodulin. Neural networks, such as the enteric nervous system (ENS) and vagus nerve also convey information within the gut-brain axis. Emerging evidence suggests the human gut microbiota, a complex ecosystem residing in the gastrointestinal tract (GIT), may influence weight-gain through several inter-dependent pathways including energy harvesting, short-chain fatty-acids (SCFA) signalling, behaviour modifications, controlling satiety and modulating inflammatory responses within the host. Hence, the gut-brain axis, the microbiota and the link between these elements and the role each plays in either promoting or regulating energy and thereby contributing to obesity will be explored in this review.

## Obesity: an increasing problem

Obesity is one of the most rapidly escalating epidemics faced by global public-health systems, in particular, those belonging to developed Westernised societies, such as Australia, United States, United Kingdom, and Canada. In the 1970s, overweight and obesity were uncommon with less than 15% of Australians being described in this category (Hayes et al., 2017). By 1995, the rate of overweight and obesity had increased to approximately 20% (Tolhurst et al., 2016; Hayes et al., 2017). Australia now possesses one of the highest incidence of overweight and obesity worldwide, affecting 63.4% of adults and 29.5% of people aged less than seventeen (Grima and Dixon, 2013; Tolhurst et al., 2016). Additionally, 44.5% of adults and between 70.1 and 91.7% of people 17 or under do not meet the minimum daily physical activity requirements and approximately 40% of the nation acquire their daily energy intake from “junk” food, which is described as a Westernised-diet high in both saturated and *trans* fats and simple carbohydrates and, therefore, hyper-caloric (Tolhurst et al., 2016). Obesity occurs when there is increased fat deposition following an imbalance between energy consumption and expenditure, where consumption exceeds expenditure. Extending from this simplistic definition, obesity is a consequence of multifaceted interactions among genetic, environmental, socio-economic, psychological, and dietary factors, thus making obesity a complex disease to understand and combat (Moran and Shanahan, 2014; Bauer et al., 2016).

Obesity is characterised by the presence of parameters indicating increased adiposity, low-grade inflammation, dysbiosis, increased neurogenic tone and hormonal imbalances (Buhmann et al., 2014; Moran and Shanahan, 2014; Bauer et al., 2016). These obesogenic factors give rise to comorbidities (Table 1), which in turn increase morbidity and mortality. Therefore, obesity determinants, as well as the associated costs, which are in excess of $8 billion per year in Australia alone, and unsuccessful non-invasive treatment interventions have resulted in an increase in research aimed at improving weight-loss approaches (Grima and Dixon, 2013; Buhmann et al., 2014). Currently, bariatric surgeries such as Roux-en-Y gastric bypass, laparoscopic sleeve gastrectomy and laparoscopic adjustable gastric banding, are the most effective treatments in increasing and sustaining long-term weight loss. However, it is relatively unknown why bariatric surgeries are successful. It is suggested that changes in the systemic and local concentrations of gut-derived peptides and the altered responses that are subsequently generated at the sites of action, in addition to changes in vagal firing and, therefore, signalling to the brain may be the key to understanding the success of bariatric surgeries (Santo et al., 2016; Yavuz et al., 2017). Consequently, a large degree of knowledge regarding the interplay between the central nervous system (CNS) and the gastrointestinal tract (GIT), and more recently the gut microbiota, with regard to energy homeostasis has been generated. Hence this review will focus on exploring the link between the gut-brain-microbiota axis and the role each aspect of this axis plays in either promoting or regulating energy, thus contributing to the obesogenic state.

**Table 1:** Overweight and obesity comorbidities in different physiological systems.

**Physiological system**	**Comorbidities**	**References**
Cardiovascular	Stroke Myocardial infarction Angina Coronary heart disease Cardiac failure Hypertension Deep vein thrombosis Pulmonary embolism Dyslipidaemia	Wilson et al., 2002; Stein et al., 2011; Global Burden of Metabolic Risk Factors for Chronic Diseases Collaboration et al., 2014; Writing Group et al., 2014; Klovaite et al., 2015; Aune et al., 2016
Gastrointestinal	Non-alcoholic fatty liver disease Gallbladder and pancreatic disease Gastro-oesophageal reflux disease Liver, colorectal, oesophageal, gallbladder and pancreatic cancers	Chen et al., 2012; Eslick, 2012; Stinton and Shaffer, 2012; DiBaise and Foxx-Orenstein, 2013
Endocrine	Non-insulin dependent diabetes mellitus Gestational diabetes mellitus Polycystic ovary syndrome	Flegal et al., 2007; Arendas et al., 2008; Yang et al., 2008
Genitourinary	Chronic kidney disease/chronic renal failure Kidney stones Renal and prostate cancers Urinary incontinence Erectile dysfunction Buried penis	Bump et al., 1992; Esposito et al., 2004; Ejerblad et al., 2006; Flegal et al., 2007; Polednak, 2008; Munkhaugen et al., 2009; Pestana et al., 2009; Stinton and Shaffer, 2012; Grima and Dixon, 2013
Pulmonary	Obstructive sleep apnoea Obesity hypoventilation syndrome Asthma Chronic obstructive pulmonary disease	Guerra et al., 2002; Steuten et al., 2006; Eisner et al., 2007; O'Donnell et al., 2014
Musculoskeletal	Osteoarthritis Spinal disc disorders and lower back pain Tendons, fascia and cartilage disorders Foot pain Impaired mobility	Molenaar et al., 2008; Tukker et al., 2009; McAdams DeMarco et al., 2011; Grima and Dixon, 2013
Reproductive	Menstrual disorders Pregnancy complications, such as miscarriage and intrauterine foetal death Birth defects Infertility Breast (post-menopause), endometrial and ovarian cancers	Bianchini et al., 2002; Arendas et al., 2008; Polednak, 2008; Grima and Dixon, 2013
Mental/Psychological	Dementia Depression Eating disorders Reduced health-related quality of life Psychosocial stigma and poor self esteem	Beydoun et al., 2008; Molenaar et al., 2008; Grima and Dixon, 2013; Hilbert et al., 2014
Integumentary	Increased sweat gland activity Impaired epidermal barrier repair Striae Cellulitis Hyperpigmentation Intertrigo Lymphoedema	Löffler et al., 2002; Yosipovitch et al., 2004, 2007
Immune	Disruption of lymphoid tissue integrity Changes in leukocyte development, phenotypes and activity Decreased immunity from infection Decreased efficacy of vaccines Increased pro-inflammatory markers, such as IL6 and TNFα	Ghanim et al., 2004; Bremer et al., 2011; Kanneganti and Dixit, 2012; Sheridan et al., 2012

The CNS, in particular, the brain, has the elaborate task of interpreting continuous information provided to it by neural networks and chemical messengers with respect to the body's energy state. It uses this information to initiate an appropriate reaction to maintain homeostasis. These signals vary throughout time and the responses alter depending on what type of food has been ingested. Although foodstuffs are first encountered by the oral microbiome, the GIT remains one of the primary sites where they are first sampled. Therefore, the gut becomes responsible for generating the majority of inputs communicated to the CNS regarding the content and size of a meal, thus establishing a complex bi-directional communication system, referred to as the gut-brain axis (Bauer et al., 2016; Gribble and Reimann, 2016).

## The gut-brain axis: connections from the gut to the brain

The gut-brain axis is a complex neurohumoral communication network imperative for maintaining metabolic homeostasis. It is comprised of the CNS, enteric nervous system (ENS), the autonomic nervous system (ANS) and its associated sympathetic and parasympathetic branches, neuroendocrine and immunological systems, in addition to the gut microbiota, which will be discussed below (Grenham et al., 2011). Axis communication is formed through sensory information being converted into neural, hormonal and immunological signals, which are relayed back and forth from the CNS to the gut and vice versa (Mayer et al., 2015). Whilst there is increasing evidence that changes in intestinal immune-signalling convey shifts in gut-facilitated energy homeostasis, the majority of recognised axial effects on energy homeostasis are a consequence of neural and hormonal gut-derived signals, as the GIT possesses over 500 million neurons and is capable of producing an array of hormones (Monje, 2017). Hence, due to the large degree of innervation supplying the GIT, preabsorptive foodstuffs can initiate signals to the CNS regarding macronutrient content and caloric value through individualised nutrient-specific sensory mechanisms located throughout the GIT (Hamr et al., 2015). These signals are subsequently conveyed to various regions of the brain, such as the brainstem and hypothalamus. The higher-order processing of these centres consequently initiates a series of reactions that result in both acute and chronic deviations in energy consumption and expenditure, thus maintaining metabolic homeostasis pre- and post-prandial (Buhmann et al., 2014).

**Figure 1:**
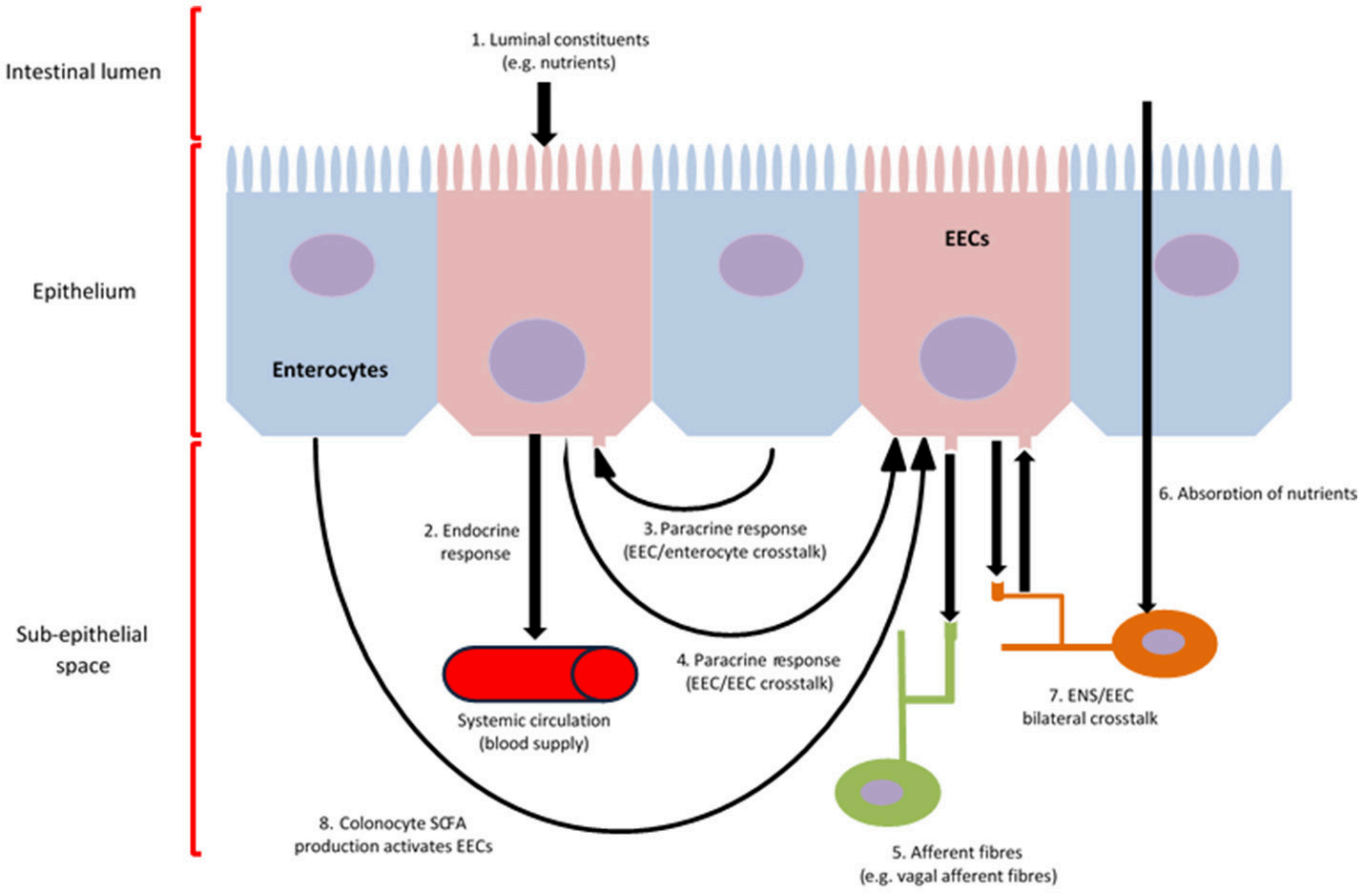
EEC function and communication. Intracellular metabolism and activation of chemoreceptors located on the apical cell membrane of EECs, result in calcium influx, which induces the synthesis and release of gut hormones into the sub-epithelial space (1, 4) (Psichas et al., 2015). Various gut-derived hormones are synthesised and secreted in response to luminal constituents and released from EECs systemically to induce an effect on various tissues throughout the body, such as the brain, via, metabolic, local, paracrine (3) and/or endocrine (2) action, as well as the activation of afferent neurons innervating the GIT wall (5, 6, 7, 8) (Psichas et al., 2015). Further, EEC/ENS crosstalk can result from the direct absorption of nutrients through the intestine (7). The production of SCFA by the microbiome, which can be subsequently utilised by colonocytes as an energy source, can activate EECs, thus contributing to gut-brain activation (8).

Gut hormones are released by enteroendocrine cells (EECs), which initiate the majority of signalling and communication within the gut-brain axis in response to preabsorptive nutrients. These cells are located throughout the epithelium of the GIT, with many containing an apical cell membrane covered in microvilli, which open to and directly contact the luminal contents (Gribble and Reimann, 2016). An overview of EEC function is provided below (Figure 1).

The bulk of digestion and nutrient absorption occurs within the stomach and small intestine. Therefore, these organs are highly innervated as they are the primary sites responsible for nutrient-sensing. This dense area of innervation originates from the vagal and splanchnic nerves (Bauer et al., 2016). Here the quantity of afferent fibres outnumbers the quantity of efferent fibres, indicating a fundamental role of neuronal gut-to-brain signalling (Prechtl and Powley, 1990; Berthoud et al., 1995). Vagal fibres in particular, extend into the lamina propria of the intestinal villi, terminate at the basolateral cell membrane of EECs and express receptors for gut hormones such as ghrelin, leptin, cholecystokinin (CCK), glucagon-like peptide 1 (GLP1), and peptide YY_3−36_ (PYY), thus leading to receptor activation and subsequent neuronal stimulation (Dockray, 2013). Additionally, ENS neurons, which possess receptors for various gut hormones, may indirectly activate vagal and spinal afferents (Amato et al., 2010; Richards et al., 2014). Whilst the ENS controls intestinal function locally via reflex actions, it cannot be dismissed from playing a role in transmitting nutrient-derived signals to vagal afferents, thus contributing to the gut-brain axis (Costa et al., 2000; Sayegh et al., 2004). Intrinsic ENS neurons are proximally located to both EECs and various afferent nerve terminals; stimulated by intestinal nutrient infusion; and stimulate vagal afferent fibres in the gut (Sayegh et al., 2004; Ritter, 2011). Whilst the exact mechanisms have not been completely elucidated and the notion that the ENS can function independently of CNS involvement is still favoured, it is clear from these studies that the gut-brain neuronal-signalling axis is initiated by nutrient-induced gut hormone secretion.

**Figure 2:**
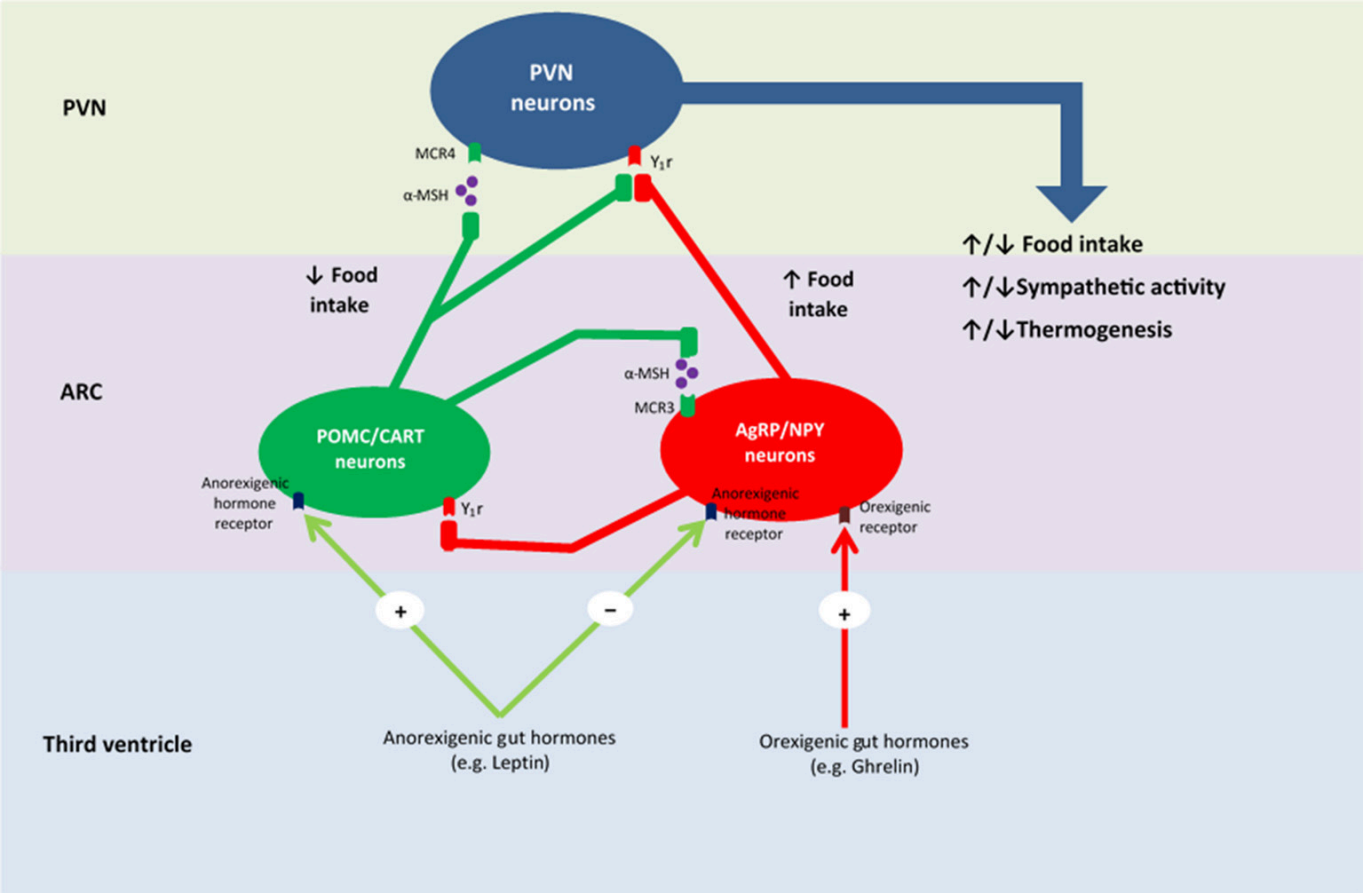
Proposed mechanism of energy homeostasis within the hypothalamus. PVN, paraventricular nucleus; ARC, arcuate nucleus; MCR4, melanocortin 4 receptor; α-MSH, α- melanocortin-stimulating hormone; MCR3, melanocortin 3 receptor; Y1r, neuropeptide Y receptor type 1; POMC, pro-opiomelanocortin; CART, cocaine- and amphetamine-regulated transcript; NPY, neuropeptide Y; AgRP, agouti-related protein.

Upon food consumption, sensory information is conveyed from the gastrointestinal vagal and/or somatosensory (spinal) afferent fibres to the nucleus tractus solitarius (NTS). More specifically, vagal afferents converge in the NTS of the dorsal vagal complex within the brainstem, and somatosensory afferents synapse with neurons in the posteromarginal nucleus of the spinal dorsal horn, which then project to the NTS (Zittel et al., 1994; Schwartz et al., 2000). The NTS, in turn, integrates and carries these gut-derived signals to the hypothalamus (Craig, 1996; Schwartz et al., 2000). Using c-Fos—a marker used to represent increased neuronal activity—Zittel et al. (1994) demonstrated that its expression in the NTS increased upon nutrient infusion within the gut, whilst high-dose capsaicin treatment, which acts as a neurotoxin, decreased c-Fos expression and blocked gut-brain vagal communication (Mönnikes et al., 1997). Additionally, Campos et al. (2013) reported that NTS neurons were stimulated by vagal afferents by the activation of *n*-methyl-D-aspartate (NMDA) receptors in afferent terminals, which subsequently led to neurotransmitter release via phosphorylation of extracellular signal-related kinases 1/2 and synapsin 1. Babic et al. (2009) established that other NTS neurons are activated via vagal afferents stimulating pro-opiomelanocortin (POMC) and catecholaminergic neurons. These NTS neurons have been linked to contributing to satiety via signalling melanocortin receptors within the hypothalamus (Figure 2). Interestingly, a deficiency in melanocortin-receptor 4 has been demonstrated to contribute to obesity (Farooqi et al., 2003). Whilst, more studies in this area are needed to confirm the exact mechanisms as to how these different receptors and neurons interact, it may lead to a potential and more advanced understanding of how NTS subset populations contribute to energy homeostasis. Furthermore, the findings implicating POMC and catecholaminergic neuron stimulation via a vagal pathway, as well the presence of NMDA receptors within the NTS, may assist in understanding the pathways that link food consumption with behavior modifications, given that these neurons release neurotransmitters, such as dopamine, which are linked to reward, arousal, motivation and emotion.

NTS neurons project and terminate at several higher-order centres of the brain, including the melanocortin system incorporating the hypothalamus (Suzuki et al., 2012). The hypothalamus performs the fundamental role of integrating peripheral humoral signals that transduce information regarding nutrient consumption and energy expenditure, as well information relayed from the NTS and other superior regions of the brain (Bauer et al., 2016). Of particular importance with respect to feeding behaviour and energy homeostasis are the arcuate (ARC), paraventricular, ventromedial and dorsomedial nuclei, as well as the lateral hypothalamic area (Cone et al., 2001; Suzuki et al., 2012). These hypothalamic areas are unified by circuits that regulate energy homeostasis. However, the majority of studies have focused on the ARC and its role in relation to energy homeostasis (Suzuki et al., 2012; Buhmann et al., 2014). Hence, further studies are required to explore the exact role that the residual hypothalamic regions convey in relation to feeding behaviour and energy homeostasis. Nonetheless, it is clear that nutrient-sensing occurs in the gut and triggers an array of neural and/or humoral pathways that contribute to the bi-directional communication system referred to as the gut-brain axis, which subsequently regulates energy balance.

The ARC responds to peripheral and central appetite signals via tightly-regulated neurotransmitter release from two separate neuronal populations, POMC and agouti-related protein (AgRP) neurons. AgRP neurons, located in the medial ARC, release the inhibitory neurotransmitters AgRP and neuropeptide Y (NPY) (Cone et al., 2001; Suzuki et al., 2012). These neurotransmitters act to stimulate hunger and appetite, as well as decrease energy expenditure, thus contributing to excessive food consumption and weight-gain (Dryden et al., 1995; Ollmann et al., 1997; Enriori et al., 2007). POMC neurons in the lateral ARC release POMC, which stimulates the release of α- melanocortin-stimulating hormone (α-MSH) and cocaine-and-amphetamine-regulated transcript (Suzuki et al., 2012). These neurotransmitters are antagonistic of AgRP and NPY and act by decreasing appetite and hunger, thus inhibiting food intake, as well as increasing energy expenditure, thus contributing to weight-loss (Cowley et al., 2001; Nakhate et al., 2011). Therefore, energy homeostasis involves a delicate balance between these two neuronal populations.

Adding to the complexity of hypothalamic function is that gut hormones, which increase in concentration post-prandial, have direct access to the ARC (van der Kooy, 1984). The ARC and the NTS are proximally located in an area of the brain that possesses an incomplete barrier, thus contributing to a leaky blood-brain-barrier (Bauer et al., 2016). This area is referred to as the area postrema (AP). Lesioning the AP and vagotomy diminish the effects of multiple gut hormones, thus indicating that gut hormones directly influence these brain regions once released systemically (van der Kooy, 1984; Date et al., 2002). Batterham et al. (2002) demonstrated an example of this by peripheral injection of PYY into the general circulation. PYY binds to Y_2_ receptor, which Batterham et al. (2002) localised to the ARC by demonstrating an increase in c-FOS immunoreactivity. Additionally, Seeley et al. (1994) established that chronic decerebrate rats, who only had the brainstem intact, had suppressed food intake and increased energy expenditure in response to intestinal nutrient infusion. This early 1990s study provides support for latter findings that the NTS may receive gut-derived signals systemically due to a leaky blood-brain-barrier (Bauer et al., 2016). It is also interesting that these rats had increased energy expenditure, given their decerebrate state (Seeley et al., 1994). This may implicate the brainstem in regulating energy expenditure through its role as a motor output cortex. More elaborate studies combining motor output with intestinal sampling are warranted. Additionally, this may assist in providing a link to dietary intake and exercise and the role that each of these factors play in relation to energy homeostasis.

## Gut hormones and the role of the gut-brain axis in energy homeostasis

Whilst neural connection has been explored, it is also imperative to mention the stomach's role in nutrient intake. The stomach is one of the first organs to generate a feedback signal to the melanocortin system. When food enters the GIT, as a bolus, the stomach becomes stretched, triggering a feedback-loop to the brain to cease eating. The gastric emptying of foodstuffs into the duodenum occurs once the pyloric sphincter is summoned to relax. Once the nutrients enter the duodenum, the rate of emptying decreases, thus augmenting gastric distension and limiting the amount of food consumed.

The rate of gastric emptying is decreased by vagal activation and the release of gut hormones, such as CCK, PYY, and GLP1 (Cooke and Clark, 1976; Talsania et al., 2005; Suzuki et al., 2012). This negative feedback signal was demonstrated by Davis and Smith (1990), who established that food intake diminishes within 6 min of feeding in fasting re-fed rats, thus preventing excessive food consumption. Phillips and Powley (Phillips and Powley, 1996) demonstrated in a rat model that stomach distension induces a feedback signal to cease excessive ingestion in less than 3 min rather than the content of the food by occluding the pyloric sphincter to prevent gastric emptying and by using saline in lieu of foodstuffs. These studies indicate that neurons supplying the stomach express mechanoreceptors, which are activated by stomach distension (stretch), contribute to relaying a limited information to the brain and provide limited assistance in nutrient-sensing and long-term energy homeostasis (Bauer et al., 2016). Emerging evidence reveals that taste receptors are found in the stomach and may contradict previous studies with regard to the type of sensory information relayed to the CNS and how the CNS integrates this information and conveys it to the rest of the gut (Young et al., 2009; Depoortere, 2014). In contrast, historical studies demonstrating that sham feeding—a process where foodstuffs that enter the stomach do not reach the small intestine and are bypassed directly to the colon or removed directly from the stomach—is inhibited through intestinal nutrient infusion, thus demonstrating that nutrients in the intestine can suppress food intake irrespective of gastric emptying and relay information to the gut-brain axis via neurohormoral mechanisms (Gibbs et al., 1981; Reidelberger et al., 1983). Hence, the role of gut hormones in relation to the control of food intake with regard to energy homeostasis will be explored. Since the effects of glucagon, insulin, leptin and ghrelin are extensive and well documented elsewhere, they will not be revised in this review and their functions are summarised in Table 2 (Sakata and Sakai, 2010; Dimitriadis et al., 2011; Jones et al., 2012; Pan et al., 2014).

**Table 2:** The sites of production, functions and systemic serum concentrations of different gut hormones during obesity and post-bariatric surgeries.

**Hormone**	**EEC type**	**Primary location**	**Function**	**Systemic concentration**		**References**
				**Obesity**	**Post-bariatric surgeries**	
CCK	I-cell	Duodenum Jejunum	Gastric emptying ↓ Pancreatic secretion ↑ Gallbladder contraction↑ Satiety ↑	Unclear	No change	Gibbs et al., 1973; van der Kooy, 1984; Calingasan et al., 1992; Lieverse et al., 1995; Kopin et al., 1999; Di Francesco et al., 2005; Cheung et al., 2009; Rasmussen et al., 2012; Duca and Yue, 2014; Dixon et al., 2015; Münzberg et al., 2015
GLP1	L-cell	Ileum	Insulin secretion ↑ β-cell proliferation ↑ β-cell gene expression ↑ Satiety ↑ Gastric acid secretion ↓ Gastric emptying ↓ Apoptosis ↓	↓	↑	Elliott et al., 1993; Deacon et al., 1995; Larsen et al., 1997; Yamato et al., 1997; Edwards et al., 1999; Villanueva-Peñacarrillo et al., 2001; Vilsbøll et al., 2003; Baggio et al., 2004; Abbott et al., 2005; Adam and Westerterp-Plantenga, 2005; Cani et al., 2005; Holst, 2007; Li et al., 2009; Pocai et al., 2009; Rüttimann et al., 2009; Suzuki et al., 2012; Wang et al., 2012; Zhang and Ritter, 2012; Mumphrey et al., 2013; Ohlsson et al., 2014; Richards et al., 2014; Dixon et al., 2015; Kuhre et al., 2015; Münzberg et al., 2015; Svendsen et al., 2015; Graaf et al., 2016
PYY	L-cell	Ileum	Gastric acid secretion ↓ Pancreatic and intestinal secretion ↓ Gastrointestinal motility↓ Satiety ↑	↓	↑	Adrian et al., 1985; Fu-Cheng et al., 1997; Batterham et al., 2002, 2003a, 2006; Abbott et al., 2005; Di Francesco et al., 2005; Koda et al., 2005; Korner et al., 2005; Stock et al., 2005; Talsania et al., 2005; le Roux et al., 2006; Oesch et al., 2006; Pyarokhil et al., 2012; El-Salhy et al., 2013; Dixon et al., 2015; Münzberg et al., 2015
PP	F-cell	Pancreas	Gastric emptying ↓ Leptin levels (white adipose tissue) ↓ Satiety ↑	↓	No change	Adrian et al., 1976, 1985; Schwartz et al., 1978; Lassmann et al., 1980; Clark et al., 1984; Berntson et al., 1993; Parker and Herzog, 1999; Batterham et al., 2003b; Balasubramaniam et al., 2006; Lin et al., 2009; Habib et al., 2012; Kohno and Yada, 2012; Pyarokhil et al., 2012; Dixon et al., 2015; Khandekar et al., 2015
Oxyntomodulin	L-cell	Ileum	Gastric emptying ↓ Gastric acid secretion ↓ Blood glucose ↓ Insulin secretion ↑ Energy expenditure ↑ Satiety ↑	↓	Unclear	Anini et al., 1999; Dakin et al., 2002, 2004; Cohen et al., 2003; Baggio et al., 2004; Wynne et al., 2005, 2006; Maida et al., 2008; Druce et al., 2009; Pocai et al., 2009; Habib et al., 2012; Pocai, 2014; Meek et al., 2016
Serotonin	Enterochromaffin cell	Various sites	Gut contraction ↑ Gut motility ↑ Emesis ↑/↓ Nitric oxide production ↑ Absorption ↑ Various neurological and psychological functions	Unclear	Unclear	Halford et al., 1997; Savastano et al., 2007; Lam et al., 2008; Bertrand and Bertrand, 2010; Bertrand et al., 2011; Daly et al., 2011; Halford and Harrold, 2012; Suzuki et al., 2012; Mumphrey et al., 2013; Crane et al., 2015; Dixon et al., 2015; Hall, 2015; Münzberg et al., 2015; Stiedl et al., 2015; Palamiuc et al., 2017
GIP	K-cell	Duodenum Jejunum	Insulin secretion ↑ β-cell proliferation ↑ Satiety ↑ Lipoprotein lipase activity ↑ Fat deposition ↑ Fatty acid synthesis ↑ Bone formation ↑ Apoptosis ↓	↑	Unclear	Elliott et al., 1993; Meier et al., 2001; Stock et al., 2005; Laferrère et al., 2008; Mentlein, 2009; Mieczkowska et al., 2013; Cho et al., 2015; Meek et al., 2016
Gastrins	G-cell	Stomach	Gastric acid secretion ↑ Parietal cell maturation ↑ Fundal growth ↑ Zymogen secretion ↑ Stomach contractions ↑ Satiety ↑ Gastric emptying ↓ Pancreatic secretions ↑ Gallbladder emptying ↑	Unclear	Unclear	Valenzuela et al., 1976; Kazumori et al., 2001; Nørsett et al., 2008, 2011; Dimaline and Varro, 2014; Dixon et al., 2015; Hall, 2015; Münzberg et al., 2015
Histamine	Enterochromaffin cell	Various sites	Various neurological functions Various immunological functions Vasodilatation ↑ Gastric acid secretion ↑ Serotonin release ↑/↓	Unclear	Unclear	Powell, 1976; Jutel et al., 2001; Parmentier et al., 2002; Li et al., 2003; Panula et al., 2015; Yuan et al., 2017
Neurotensin	N-cell	Jejunum	Various neurological and psychological functions Satiety ↑ Gastric emptying ↓ Gastric acid secretion ↓ Energy expenditure ↓ Fat absorption ↑ Smooth muscle contraction ↑/↓	Unclear	Unclear	Blackburn et al., 1980; Katz et al., 2004; Fawaz et al., 2009; Feifel et al., 2010; Kalafatakis and Triantafyllou, 2011; Dirksen et al., 2013; Grunddal et al., 2016; Li et al., 2016
Secretin	S-cell	Duodenum Jejunum	Gastric acid secretion ↓ Bicarbonate production ↑ Bile production ↑ Gallbladder contraction ↑ pH ↑ Water secretion ↑ Insulin secretion ↑	↓	↑	Boyer and Bloomer, 1974; Gray et al., 1988; Chu et al., 2007; Cheng et al., 2011; Wang et al., 2015; Meek et al., 2016
Glucagon	α-cell	Pancreas	Blood glucose ↑ Energy expenditure ↑	↑	↓	Billington et al., 1991; Dicker et al., 1998; Korner et al., 2006; Swarbrick et al., 2008; Ortega et al., 2011; Jones et al., 2012; Dixon et al., 2015; Münzberg et al., 2015
Amylin	β-cell	Pancreas	Gastric emptying ↓ Gastric acid secretion ↓ Postprandial glucose elevation ↓	↑	↓	Higham et al., 2000; Ratner et al., 2004; Dixon et al., 2015; Hay et al., 2015; Münzberg et al., 2015
Ghrelin	A-like cells	Stomach Duodenum	Growth-hormone secretion ↑ Gastric motility ↑ Vasodilatation ↑ Cardiac contractility ↑ Hunger ↑	↓	Unclear	Kojima et al., 1999; Bedendi et al., 2003; Korner et al., 2005; Stock et al., 2005; Sakata and Sakai, 2010; Mumphrey et al., 2013; Dixon et al., 2015; Münzberg et al., 2015; Yavuz et al., 2017
Insulin	β-cell	Pancreas	Absorption ↑ Glycogen synthesis ↑ Blood glucose ↓ Lipid synthesis ↑ Lipolysis ↓ Proteolysis ↓ Autophagy ↓	↑	↓	Korner et al., 2006; Shak et al., 2008; Swarbrick et al., 2008; Dimitriadis et al., 2011; Ortega et al., 2011; Kleinridders et al., 2014; Dixon et al., 2015; Pankov, 2016
Leptin	Non-EEC Adipocytes	White adipose tissue	Hunger ↓ α-MSH secretion ↑ Energy expenditure ↑ NPY & AgRP action ↓ Endocannabinoid expression ↓	↑	↓	Elias et al., 1999; Cowley et al., 2001; Ravinet Trillou et al., 2004; Korner et al., 2005; Enriori et al., 2007; Liu et al., 2011; Pan et al., 2014; Dixon et al., 2015; Münzberg et al., 2015; Yavuz et al., 2017

### Cholecystokinin

CCK was the initial gut hormone to be implicated in appetite control and was shown to be secreted post-prandial from EECs within the duodenum and jejunum (Gibbs et al., 1973). Its release is stimulated by fat and protein ingestion and its concentration augments within 15 min post-prandial (Lieverse et al., 1995; Buhmann et al., 2014). CCK possesses a short half-life of few minutes and consequently has limited time to induce its effects by acting upon CCK-1 and CCK-2 receptors located throughout tissues of GIT and the CNS, including the vagal nerve, NTS and hypothalamus (Buhmann et al., 2014; Lo et al., 2014). CCK increases gallbladder and gastrointestinal motility and secretion, in addition to playing a significant role in initiating the gut-brain axis to control food intake, energy expenditure and glucose utilisation (Cheung et al., 2009; Suzuki et al., 2012). Peripheral administration of CCK in animal studies regulates food intake in a dose-dependent manner and administration of CCK-1 receptor antagonists in conjunction with fatty-acid and protein consumption impedes the stimulation of vagal afferents lining the small intestine as well as the regulatory effects of CCK on food ingestion (Calingasan et al., 1992; Cox et al., 1996; Duca and Yue, 2014). Hence, these studies implicate CCK as a specific mediator of fat and protein satiation. Additionally, repeated doses of CCK into the systemic circulation and sporadic CCK infusion during feeding, initially decreases the amount of foodstuffs ingested, but over time, a tolerance to CCK develops and the quantity and frequency of ingestion increases (Kopin et al., 1999; Buhmann et al., 2014). This desensitising effect may explain the failed attempts to utilise CCK-derivatives, such as GI 181771X, as an effective weight-loss treatment (Castillo et al., 2004; Kim et al., 2011).

CCK administration conveys glucose-regulating effects, via increased vagal firing, which in turn induces the NDMA neurons of the NTS to increase hepatic vagal firing to signal the liver to decrease gluconeogenesis (Rasmussen et al., 2012). When rats are placed on a high-fat, high-carbohydrate diet, they develop CCK-resistance in response to the increased levels (Daly et al., 2011). The exact mechanisms how CCK administers its glucoregulatory effects and how CCK resistance develops remain unclear. However, these findings may provide an explanation as to why CCK-derivatives induce pancreatitis and contribute to developing an impaired utilisation of glucose. Further studies outlining the molecular physiology that CCK conveys on other organs, such as the liver requires further research.

### Glucagon-like peptide 1 (GLP1)

GLP1 is a neuropeptide released predominantly from EECs of the ileum and colon in response to carbohydrate, lipid and/or protein ingestion (Elliott et al., 1993; Adam and Westerterp-Plantenga, 2005). It is synthesised by post-translational processing of the preproglucagon gene in the CNS and the GIT, exerting its effects via activating the GLP1 receptor, which is a type of GPCR expressed extensively throughout the CNS, GIT, and pancreas (Larsen et al., 1997; Yamato et al., 1997). Systemic and central GLP1 administration stimulates satiety centres in the brain, in particular, the ARC, paraventricular, NTS and AP to decrease hunger (Larsen et al., 1997; Abbott et al., 2005). Hence GLP1 is considered to be a pivotal factor leading to satiation. It is synthesised and released within 15 min post-prandial, which is intriguing given that intestinal L-cells are located distally in the ileum (Elliott et al., 1993; Bauer et al., 2016). Therefore, GLP1 release may be a reflex response involving vagal fibres located within the duodenum, given that these fibres are involved in early nutrient-sensing. Whilst this hypothesis has yet to be validated, recent studies have demonstrated the presence of GLP1-secreting EECs within the duodenum, indicating that GLP1 release may occur in two stages or in response to the hypothesised reflex (Svendsen et al., 2015). Whilst more elaborate studies involving nutrient infusion into sections of the small intestine are needed in order to determine the exact site/s GLP1 is secreted from, what is clear is that its release is relative to the energy intake and to all types of macromolecules to induce satiety.

GLP1 is a powerful incretin (a blood glucose-regulating peptide) that stimulates the GLP1 receptor of pancreatic β-cells to release insulin (Buhmann et al., 2014). Additionally, enhanced levels of GLP1 upregulate pancreatic β-cell gene expression of insulin promoter factor 1, thus promoting their development and impeding their apoptosis, which in turn contributes to improved glucose utilisation within the body (Villanueva-Peñacarrillo et al., 2001; Suzuki et al., 2012). Finally, GLP1 decreases the rate of gastric emptying into the duodenum and hinders gastric acid secretion, which in turn increases gastric distension, limits excessive food consumption, enhances satiety and positively contributes to energy homeostasis (Edwards et al., 1999).

Whilst the effects conveyed by GLP1 are potent, they are often brief as GLP1 is vulnerable to rapid degradation and inactivation through the catalytic function of dipeptidyl peptidase IV (DPPIV) (Deacon et al., 1995; Holst, 2007). Only 10% of intestinal-derived GLP1 reach the systemic circulation, indicating that it conveys its effects in a paracrine manner (Vilsbøll et al., 2003; Holst, 2007; Kuhre et al., 2015). Additionally, peripheral administration of GLP1 in conjunction with the removal of the vagus nerve impedes the effects of GLP1, whilst intravascular infusion of GLP1 continues to convey its effects in the presence of vagotomy and/or high doses of the neurotoxin capsaicin (Rüttimann et al., 2009; Zhang and Ritter, 2012). This indicates that the GLP1 receptor is located within the brain and that GLP1 may elicit higher-order functions, as a neurotransmitter, which are yet to be determined. Whilst GLP1 stimulates specific regions of the brain, such as the brainstem, to enhance motor output and/or thermogenesis, further studies are needed to determine the mechanism/s involving GLP1 and higher-order neural function with regard to energy homeostasis and food behaviour patterns (Li et al., 2009; Graaf et al., 2016). Additionally, Ohlsson et al. (Ohlsson et al., 2014) demonstrated that GLP1 concentrations rise rapidly post-prandial within the lymph and that these concentrations are sustained for longer intervals, as DPPIV is expressed at lower concentrations within the lymph than the general circulation. Hence, this may provide another pathway as to how GLP1 exerts its effects centrally and peripherally, as well as the role it may possess with respect to immune-signalling and the inflammatory state associated with obesity. Either way, future studies that aim to extend GLP1 function and/or mimic its function through the use of GLP1 receptor agonists, such as exenatide, may offer promise in relation to increasing satiety and regulating energy homeostasis and, therefore, treating obesity.

### Peptide YY_3−36_ (PYY)

PYY is a small peptide belonging to the pancreatic-peptide family and, like GLP1, is secreted by intestinal L-cells post-prandial (Batterham et al., 2002, 2006). It is released in response to intestinal nutrient-sensing and in volumes that reflect the amount of energy consumed (Oesch et al., 2006). PYY is secreted with 15 min post-prandial, in a manner emulating GLP1 with regard to duodenal nutrient-sensing, increased vagal-firing and/or chemically-derived reactions (Fu-Cheng et al., 1997). Unlike CCK and GLP1 whose concentrations diminish rapidly, PYY concentrations remain elevated for several hours post-prandial (Batterham et al., 2003a). Hence, PYY effects may be prolonged and exhibited in a more endocrine fashion in comparison to CCK and GLP1.

PYY is present throughout the entire GIT, from the oesophagus through to the rectum (Adrian et al., 1985). PYY binds to the Y_2_ receptor and, in turn, decreases food intake, as studies using rodents lacking this receptor and PYY knockout mice become polyphagic and, consequently, gain weight (Batterham et al., 2006; le Roux et al., 2006). Additionally, PYY elicits activation of the NTS and POMC neurons in the ARC, via peripheral and central administration, indicating the presence of Y_2_ receptors on the vagus nerve, within the NTS and in the ARC (Batterham et al., 2002, 2006; Koda et al., 2005; le Roux et al., 2006). PYY exerts its effects by inhibiting NPY neurons, as Y_2_ receptors are expressed abundantly by these neurons in the ARC and their activation consequently impedes the orexigenic effects of NPY (Dryden et al., 1995; Broberger et al., 1997). Therefore, PYY may possess a pivotal role in energy homeostasis by regulating food intake and suppressing excessive consumption in an endocrine fashion, via activation of the POMC neurons and inhibition of NPY within the melanocortin system (Bauer et al., 2016). Additionally, studies indicate that obese subjects possess lower post-prandial PYY concentrations, whilst other studies suggested that there are vast differences between healthy and obese individuals with regard to fasting PYY concentrations (Batterham et al., 2003a; Korner et al., 2005; Stock et al., 2005). Augmented PYY concentrations are associated with gastrointestinal diseases such as inflammatory bowel disease and chronic destructive pancreatitis, in addition to prolonged appetite loss (El-Salhy et al., 2013). Additionally, sustained concentrations of PYY and CCK in the elderly are concomitant with delayed gastric emptying and reduced cholecystic contractility (Di Francesco et al., 2005; Buhmann et al., 2014). The mechanism as to why this occurs remains elusive. However, increased concentrations, which assist in long-term satiety and therefore a reduced energy-intake, may be linked to malnutrition in the elderly (Di Francesco et al., 2005; Buhmann et al., 2014). Hence, further studies are needed to determine the long-term effects of raised PYY concentrations before Y_2_ receptor agonist and/or PYY derivatives can be utilised as effective anti-obesogenic treatment.

### Pancreatic polypeptide

Pancreatic polypeptide (PP) belongs to the pancreatic-peptide family and is secreted by specialised F-cells within the pancreatic islets of Langerhans (Khandekar et al., 2015). Its release, like PYY, is proportional to caloric intake, where foods high in fat trigger an increased response (Guyenet and Schwartz, 2012). It is released systemically during the preabsorptive and post-prandial state, suggesting that its secretion emulates GLP1 and PYY with regard to duodenal nutrient-sensing, increased vagal-firing and/or chemically-derived reactions (Schwartz et al., 1978; Khandekar et al., 2015). Its concentration has been demonstrated to be sustained and elevated for up to 6 h post-prandial, thus suggesting an endocrine action (Adrian et al., 1976). Furthermore, PP is released in the colon and rectum of the bovine gut, where it acts as an exocrine hormone (Pyarokhil et al., 2012). This function has not been validated in human studies, hence it is unknown if it exerts an exocrine function within the human.

PP acts upon the Y_4_ receptor within the AP, NTS, and the ARC and concurrently induces gallbladder relaxation and inhibits pancreatic secretion, as it acts as a CCK antagonist, in addition to delaying gastric emptying, which leads to a rapidity of satiety and a decreased food consumption (Parker and Herzog, 1999; Balasubramaniam et al., 2006; Lin et al., 2009). The role PP plays with respect to appetite suppression is enhanced by studies demonstrating a difference in PP concentrations in anorexic and obesogenic states, where it is increased and diminished respectively (Batterham et al., 2003b). Whilst PP is a potent appetite suppressant, a seminal study conducted by Clark et al. (1984) demonstrated that central administration of PP stimulated appetite and led to an enhanced food intake. Whilst there is conflicting evidence that does not support these findings, the findings by Clark et al. (1984) have yet to be refuted and further studies are needed to determine if central-acting PP appeases or stimulates appetite. Moreover, studies in Prader-Willi syndrome and obese patients established a diminished level of PP post-prandial in comparison to healthy individuals and that intravenous PP injection in these patients led to a significant decline in food consumption (Lassmann et al., 1980; Berntson et al., 1993). PP-overexpressing mice have an increased incidence of mortality, which is resultant of a reduction in maternal milk consumption (Kohno and Yada, 2012). Whilst this can be portrayed as extreme, it validates the potential potency of PP with respect to satiation. Further, Obinepitide (7TM Pharma), a potent synthetic analogue of PP and a Y_4_ receptor agonist, has demonstrated decreases in both food intake and weight loss (Davenport and Wright, 2014). Although its use has been reported to be well tolerated with minimal adverse side effects, trials have only been conducted for 28 days (Davenport and Wright, 2014). Hence further studies are needed to establish the chronic effects that PP may possess before Y_4_ receptor agonists and PP-derived agents are utilised as potential anti-obesogenic treatments.

### Oxyntomodulin

Oxyntomodulin is a peptide hormone secreted in response to nutrient ingestion. It is synthesised, like GLP1, by post-translational processing of the preproglucagon peptide within EECs of the gut and the CNS (Cohen et al., 2003; Baggio et al., 2004; Habib et al., 2012). Its secretion occurs concurrently with GLP1 and PYY and reaches its peak concentration within 30 min post-prandial before it is rapidly degraded by DPPIV (Anini et al., 1999; Druce et al., 2009). Oxyntomodulin binds to GLP1 receptors within the GIT, the pancreas and the ARC, to induce a decrease in gastric acid secretion and food consumption, as outlined previously in the GLP1 section of this review (Baggio et al., 2004; Dakin et al., 2004; Pocai et al., 2009). Central and peripheral oxyntomodulin administration enhances satiety and therefore decreases food consumption in rodent and human models, as well as increasing energy expenditure (Dakin et al., 2002, 2004; Cohen et al., 2003; Baggio et al., 2004; Wynne et al., 2005). Additionally, oxyntomodulin binds to glucagon receptors within the pancreas, lowers blood glucose concentrations and improves glucose utilisation (Maida et al., 2008). Whilst oxyntomodulin binds to the GLP1 receptor and is released concurrently with GLP1, the exact mechanism as to how oxyntomodulin functions as a potent incretin by binding to the glucagon receptor is largely unknown (Pocai et al., 2009; Pocai, 2014). It may act antagonistically to glucagon and induce an insulinotropic effect through a local, paracrine effect and/or it may activate higher centres of the brain via the hypothalamus. Since it has been reported to augment energy expenditure, it may also activate catecholaminergic and/or POMC neurons, as well as increase vagal activity at the site of brown fat, leading to increased thermogenesis (Dakin et al., 2002; Wynne et al., 2006; Pocai, 2014). Whilst these hypotheses are plausible, further studies are required to elucidate the mechanism/s involved in its incretin and thermogenic abilities before analogues are used in the treatment of obesity.

### Serotonin

Enterochromaffin cells, which are specialised EECs, produce and secrete gut-derived serotonin in response to food intake (Bertrand and Bertrand, 2010). Serotonin induces its effects by acting locally and systemically on various 5-HT receptors such as the 5-HT2 receptor family and the 5-HT4 receptor expressed on vagal afferent fibres and other neurons within the CNS, as well as cells within the GIT, heart, and adrenal glands (Halford and Harrold, 2012; Li et al., 2015; Stiedl et al., 2015). Serotonin analogues, such as lorcaserin, suppress appetite and decrease body weight, whilst serotonin receptor antagonists induce the contrary and increase appetite and therefore body weight (Halford et al., 1997; Savastano et al., 2007; Lam et al., 2008; Smith et al., 2010). Whilst serotonin diminishes appetite through effects on the CNS and assists in weight-loss, the contrary has been demonstrated, where animals fed a westernised diet that in turn became obese, possess increased concentrations of serotonin (Crane et al., 2015). Additionally, Crane et al. (2015) demonstrated that the inhibition of peripheral serotonin synthesis reduced obesity and metabolic dysfunction, as serotonin blunted the effects of β-adrenergic neurons supplying brown adipose tissue, which decreased thermogenesis. Furthermore, serotonin analogues have been recently withdrawn from the market due to many undesirable and severe side-effects, such as psychiatric disorders, cardiotoxicity, drug addiction and death (Onakpoya et al., 2016). Hence the effects that serotonin may convey with respect to energy homeostasis and food behaviours are complex, require substantial further investigation and are outside the scope of this review.

### Endocannabinoid system

Bioactive lipids belonging to the endocannabinoid system, such as anandamide, have been demonstrated to play a role in the gut-brain axis. These molecules are synthesised and secreted within the GIT and act upon endocannabinoid receptors, mainly cannabinoid receptors 1 and 2 (CB1/CB2), which are GPCRs within the endocannabinoid system (Moran and Shanahan, 2014; Bauer et al., 2016). CB1 is distributed abundantly throughout the CNS and the peripheral nervous system and expressed in the liver, pancreas and adipose tissue, whilst CB2 is predominantly expressed by immune cells, in addition to the brain, pancreas, and adipose tissue (Mackie, 2008). The endocannabinoid regulates various physiological functions, such as regulating gut motility and appetite, which is interesting given that the administration of exogenous cannabinoids, such marijuana, convey orexigenic effects (Mackie, 2008; Moran and Shanahan, 2014). Hence, the development of CB1 antagonists, such as rimonabant and tarabant, were utilised to induce weight-loss in obese individuals, thus demonstrating the role the endocannabinoid system plays in increasing appetite (Christensen et al., 2007; Aronne et al., 2010; Cluny et al., 2011). However, these products were withdrawn from the market, as they conveyed severe psychological side-effects such as chronic depression (Aronne et al., 2010; Moran and Shanahan, 2014).

Obesity is concomitant with enhanced endocannabinoid tone, CB1 expression, and endocannabinoid concentrations within the plasma and adipose tissues (Izzo et al., 2009; Moran and Shanahan, 2014). These findings are supported by studies demonstrating that CB1 deficient mice are resistant to diet-induced obesity and possess enhanced leptin sensitivity, which acts to inhibit hunger and increase satiety (Ravinet Trillou et al., 2004; Cluny et al., 2011). Finally, anandamide is increased during food deprivation and induces hunger by inhibiting CB1-expressing vagal afferents, which consequently blockade vagal-firing to the CNS and, therefore, may lead to a diminished effect conveyed by other gut hormones (Gómez et al., 2002; Kentish and Page, 2015). Whilst this hypothesis is yet to be confirmed, the role endocannabinoids play in relation to appetite and energy homeostasis needs to be explored, particularly since the endocannabinoid system is intricately associated with stress, memory, immune function and mood (Mackie, 2008). Hence, factors affecting these physiological and psychological functions may be associated with various food behaviours, which may be amplified in obesity. Interestingly, there is new evidence that endocannabinoids, such as anandamide, bind to Transient Receptor Potential Vanilloid 1 (TRPV1), which is located abundantly throughout most cell types in the body (Puente et al., 2011; Abdel-Salam, 2014). This is of interest as TRPV1 activation, with the use of low-dose dietary capsaicin, increases thermogenesis, suppresses appetite, improves gastrointestinal function and enhances weight-loss (Kawabata et al., 2009; Ludy et al., 2011; Ono et al., 2011; Abdel-Salam, 2014; Janssens et al., 2014). Therefore, it is evident that there is a knowledge gap with respect to the endocannabinoids and association with other physiological receptors that need to be explored and that the association of the endocannabinoids and other physiological systems are intricate and complex.

## Energy expenditure

As outlined above, obesity is a consequence of enhanced energy consumption and a decline in energy expenditure. A sedentary lifestyle and diminished energy expenditure augments weight-gain (Grima and Dixon, 2013). Energy expenditure does not just involve physical activity, it also involves thermogenesis and basal metabolic rate, even though physical activity can increase both parameters (Melanson, 2017). There are various studies demonstrating that fat-loss can occur without an increase in physical activity, indicating that energy expenditure can occur without regimented exercise and by administration of a particular intervention (Panchal et al., 2012, 2013; Owen Bryn et al., 2014). These studies retrospectively challenge previous dogma that held exercise as the “gold-standard” in terms of regulating energy and therefore fat-loss (Melanson, 2017). Additionally, the evident rise in health and fitness centres throughout many westernised-countries, such as Australia, and use of these centres, further suggests that physical activity may not lead to enhanced fat-loss without being used in conjunction with an appropriate calorie-controlled diet (Australia, 2009). Hence, gut-derived neurohumoral signals within the gut-brain axis can activate energy-regulating cortices in response to nutrient consumption to influence energy expenditure, in addition to energy consumption, thus contributing to a favourable energy balance (Bauer et al., 2016).

Peripheral and central administration of GLP1, oxyntomodulin and PYY leads to enhanced energy expenditure by increasing thermogenesis and basal metabolic rate (Dakin et al., 2002; Blouet and Schwartz, 2012). Blouet and Schwartz (2012) demonstrated that intestinal lipid-sensing activates vagal afferent fibres to enhance brown adipose tissue (BAT) thermogenesis through a CCK-dependent pathway. This suggests that a gut-brain-BAT axis may exist and is further enhanced by studies that incorporate an intervention as a treatment to induce weight-loss (Blouet and Schwartz, 2012; Panchal et al., 2012, 2013; Brown et al., 2015). An example of this is low-dose dietary capsaicin, which increases gut-derived vagal firing, augments hormone secretions, enhances sympathetic tone, and activates BAT, leading to an increased basal metabolic rate and thermogenesis, which culminates in weight-loss (Kawabata et al., 2009; Ludy et al., 2011; Ono et al., 2011; Abdel-Salam, 2014; Janssens et al., 2014). Hence, exploration of this potential axis may assist in treating obesity and its associated comorbidities, via focusing on the thermogenic properties a treatment or dietary intervention may convey.

## The gut microbiota

There is increasing evidence that the gut microbiota may influence adiposity and weight-gain through several inter-dependent pathways, including energy harvest and subsequent generation of metabolites, such as short-chain fatty-acids (SCFA), modification of host behaviour, satiety through the gut-brain axis and effects on inflammatory responses within the host (Moran and Shanahan, 2014).

**Figure 3:**
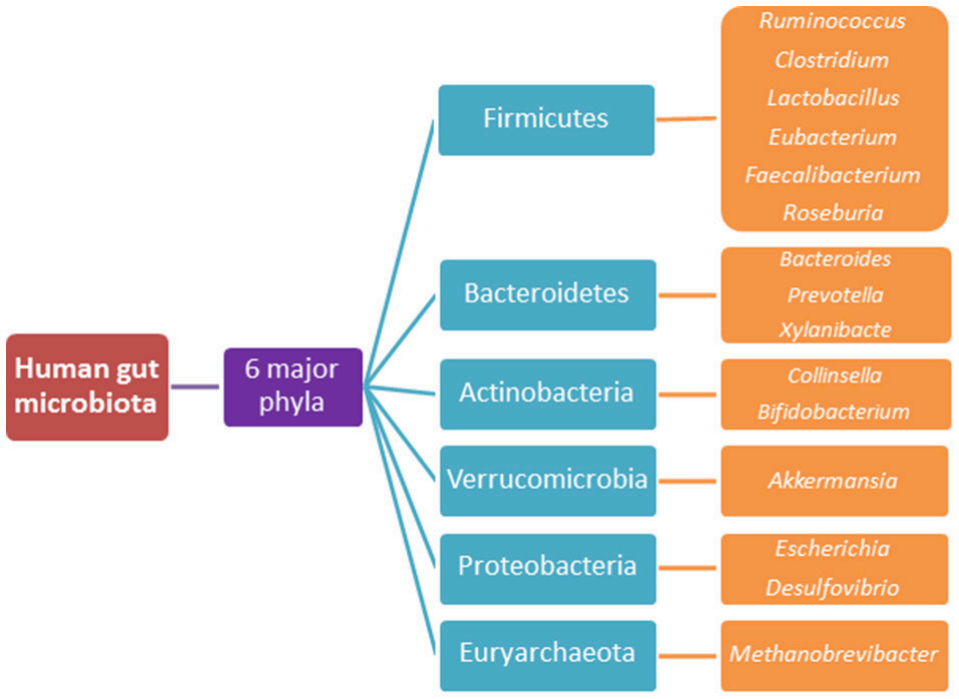
The 6 major phyla of the human gut microbiota and their predominant species.

There are in excess of 3.8–3.9 × 10^13^ bacterial cells colonising a healthy human body, a majority of which reside within the GIT and comprise the complex ecosystem referred to as the gut microbiota (Sender et al., 2016a,b). The microbial to human cell ratio has recently been revised to approximately 1.3:1 from the historical 10:1 ratio (Sender et al., 2016a,b). Nonetheless, these microbes form an intricate symbiotic relationship with the host, where the host provides a nutrient-dense environment for the microbiota and the microbiota, in turn, provides metabolic, protective and structural functions, which are not encoded for by the host's genome (Qin et al., 2010; Wang and Wang, 2016). The microbiota is thought to be comprised of more than 1000 different bacterial species (Figure 3) and this composition alters throughout the lifespan due to factors such as diet, antibiotic use, disease states, delivery method at birth and most elements that a modern lifestyle incorporates (Qin et al., 2010). Hence, its composition is not static and the changes to its structure are dynamic.

The gut microbiota conveys a vast impact on the host's metabolism and was first outlined in a seminal study conducted by Wostmann et al. (1983). Wostmann et al. (1983) demonstrated that mice lacking a microbiota (germ-free) possessed reduced adiposity, energy intake, and energy extraction from a standard rodent diet compared to conventionally-raised mice. Additionally, Wostmann et al. (1983) collated a series of studies outlining the developmental anomalies associated with germ-free rearing compared to a conventionally-raised upbringing (Table 3), which has been validated by recent studies (Heijtz et al., 2011; Cho et al., 2012; Al-Asmakh and Zadjali, 2015). This highlights the extent of the symbiotic relationship between the host and the microbiota and how microbiota modification can impact an individual's health status. Hence, manipulation of the microbiota with regard to studying the effects related to host physiology would be more meaningful than the use of germ-free rodents (Bauer et al., 2016). This is particularly valid with respect to metabolic studies, as high-fat high-carbohydrate feeding alters the microbiota's composition and diversity within a short period of time (David et al., 2014).

**Table 3:** Anatomical and physiological differences in germ-free mice compared to wild-type mice (Al-Asmakh and Zadjali, 2015).

**Characteristic**	**Difference**
Nutrition	Requirement for vitamins K and B in diet Decreased percentage body fat Normal or increased food intake
Fluid balance	Increased water intake
Metabolism	Decreased basal metabolic rate Increased secretion of amino acids and urea and little excretion of acetic acid More urea and little ammonia in intestinal contents More nitrogen in the caecal contents and faeces Elevated oxidation-reduction potential in caecal contents Altered response to anaesthetics
Circulation	Reduced total blood volume Decreased blood flow to skin, liver, lungs and GIT Increased cholesterol concentration, numbers of red blood cells and haematocrit
Liver	Reduced size Increased ferritin and cholesterol concentrations
Lungs	Thinner alveolar and capsular walls and fewer reticuloendothelial elements
Intestinal morphology	Reduction in total intestinal mass Decrease in the total surface area of the small intestine Shorter ileal villi and longer duodenal villi Shorter crypts of the small intestine Lamina propria of the small intestine thinner, with fewer cells and slower cell renewal Larger caecum with a thinner wall
Intestinal motility	Increased muscle tissue, with elongated and hypertrophied myocytes in caecum Longer transit time
Intestinal function	Enhanced absorption of vitamins and minerals, altered absorption of other macromolecules Altered enzyme content, elevated faecal concentrations of trypsin, chymotrypsin, invertase and mucin Less fatty-acids and no cyclic or branched-chain fatty acids in the intestinal content, excretion of primarily unsaturated fatty-acids
Endocrine function	Less uptake of iodine in the thyroid Decreased motor activity and hyper-responsiveness to adrenaline, noradrenaline and vasopressin
Electrolyte status	More alkaline caecal contents High concentrations of calcium and citrate and little phosphate in the urine Somewhat less sodium and low concentrations of chloride in intestinal content

De Filippo et al. (2010) compared and characterised the differences between healthy children from either a westernised-diet or a rural-diet and demonstrated distinct differences between the microbiota and consumed foodstuffs. Whilst De Filippo et al. (2010) examined children from Italy who were presumed to eat a westernised-diet, it did not consider that these children may have been reared on the “Mediterranean-diet,” which is considered as the “gold-standard” diet with respect to healthy eating (Sánchez-Villegas et al., 2016). What was clear were the differences between to the two cultures, the food consumed and, therefore, the microbiota composition. This study, as well as others, suggested that the westernised-diet and obesity, are associated with an increased ratio of bacteria belonging to the Firmicutes phylum compared to the Bacteroidetes phylum, which is reversed upon surgical and dietary interventions (Ley et al., 2006; Turnbaugh et al., 2009; De Filippo et al., 2010; Furet et al., 2010). Controversy exists with the Firmicute-to-Bacertoidetes ratio as a guide for determining the obese phenotype, as more recent studies have failed to validate this hypothesis (Zhang et al., 2009; Schwiertz et al., 2010; Finucane et al., 2014). Hence, differences at the genus and species level compared to the phyla level may be associated with changes in metabolic function (Bauer et al., 2016).

Duca et al. (2014) demonstrated distinct genera differences in germ-free rats when they were transplanted with the microbiota of either obese-prone or obese-resistant rats. Duca et al. (2014) observed 25 operational taxonomic units (OTUs) in the obese donors and recipients that were lacking entirely in the obese-resistant donors and recipients, and that these additional 25 OTUs possessed the ability to harvest extra energy from the diet. This study confirms the findings of earlier studies that suggested a possible link between obesity and a microbiome rich in genes responsible for the production of enzymes involved in energy harvesting from indigestible carbohydrates (Turnbaugh et al., 2009; Duca et al., 2014). The relationship between the microbiota composition, energy harvest and obesity is more complex than suggested, as studies have demonstrated that energy harvest and metabolite production, in the form of SCFA, are not correlated with increased weight-gain and that some SCFA may possess a beneficial role with respect to host metabolism and energy regulation (Tims et al., 2013; Bauer et al., 2016).

## Short-chain fatty-acids, energy harvest and nutrient-sensing

Approximately 60 g of dietary carbohydrates consumed daily part of the typical western diet are indigestible. The gut microbiota possesses specific glycoside hydrolases, enabling them to ferment and hydrolyse indigestible polysaccharides and produce SCFA as a metabolite within the distal colon (Moran and Shanahan, 2014). This function and subsequent generation of SCFA provides approximately 10% of the host's daily energy requirements (Schwiertz et al., 2010). Butyrate, propionate and acetate account for 95% of the biologically significant SCFA produced (Bauer et al., 2016). Colonocytes utilise butyrate as their primary energy source, the liver utilises propionate in gluconeogenesis after it has entered the portal circulation and acetate is circulated systemically to various peripheral tissues (Gao et al., 2009; Bauer et al., 2016). Butyrate production is typically attributed to Firmicutes, whilst propionate synthesis is generally associated with Bacteroidetes (Moran and Shanahan, 2014). In the obesogenic state, faeces contain an increased quantity of SCFA, in particular, propionate (Schwiertz et al., 2010). This increase in faecal SCFA content is proposed to be due to a change in microbiota composition, rather than differences in diet and/or SCFA absorption within the colon (Schwiertz et al., 2010; Rahat-Rozenbloom et al., 2014). Interestingly, Rahat-Rozenbloom et al. (2014) reported a higher proportion of Firmicutes than Bacteroidetes in the overweight cohort, which would correlate with an increase in butyrate production rather than propionate production. Hence, further studies are required to determine the differences between obese and lean individuals and why there is an increase in faecal SCFA content. Furthermore, it is interesting that SCFA, which have been demonstrated to possess anti-carcinogen properties, are increased in the obesogenic state, given that a high-fat high-carbohydrate diet is one predisposing factor attributed to the development of colorectal cancer (Bindels et al., 2012; Grima and Dixon, 2013; Irrazábal et al., 2014). Hence, further studies are required to determine the role SCFA play with respect to the development of colorectal cancer in obesity, as it could be argued that possessing a profile mirroring a slightly overweight state, where SCFA production is slightly increased, could be gastroprotective and that increased weight leading to obesity, may be detrimental.

SCFA also assist in regulating body weight, as administration of prebiotics, indigestible polysaccharides and oral and intestinal SCFA infusion lead to an enhanced metabolic state, a reduction in food consumption and a decrease in body weight (Pan et al., 2009; Bomhof et al., 2014). This occurs as prebiotics and supplements promote the growth and activity of favourable microbial species, whilst SCFA administration activates signalling pathways, resulting in an increase in gut hormone synthesis (Pan et al., 2009; Lin et al., 2012; Bomhof et al., 2014). Hence, SCFA can be considered as pivotal endogenous signalling molecules. SCFA bind and activate free fatty-acid receptors 2 and 3 (FFAR2/FFAR3), which are GPCRs located throughout the GIT, immune cells, liver and adipose tissue (Kasubuchi et al., 2015). Within the GIT, the expression of these receptors has been localised to EECs, in particular, L-cells (Kasubuchi et al., 2015). Once bound to these receptors, L-cells are signalled to synthesise and release gut hormones, such as GLP1 and PYY (Table 2). These findings are further enhanced by *in vivo* and *in vitro* studies, which have demonstrated that cell cultures and mice lacking FFAR2 and FFAR3 have impaired GLP1 and PYY release, even in the presence of SCFA infusion (Tolhurst et al., 2012). Additionally, FFAR3 is predominantly expressed within the peripheral nervous system, in particular, the ENS and ANS (Nøhr et al., 2015). Activation of these receptors within the sympathetic branch of the ANS regulate storage mechanisms within adipose tissue and influence energy expenditure via stimulating muscle and liver tissues to regulate glucose utilisation (Moran and Shanahan, 2014; Nøhr et al., 2015). Whilst the exact mechanisms involved are yet to be elucidated, it can be appreciated that the evidence conveyed thus far implicates SCFA synthesis by the microbiota to signal and stimulate the gut-brain axis.

Intestinal epithelial cells and their absorptive and secretory ability may be influenced by the gut microbiota by acting through the gut-brain axis (Bauer et al., 2016). Studies have demonstrated diminished concentrations of FFAR2 and FFAR3, increased expression of glucose transporters and sweet-taste receptors, increased sucrose consumption and absorption, and a diminished expression of the long-chain fatty-acid receptor GPCR 120 (GPR120) in germ-free mice (Duca et al., 2012; Swartz et al., 2012). GPR120 activation conveys the anti-inflammatory and insulin-sensitising effects of omega-3 fatty-acids, whilst its absence in GPR120 knock-out mice decreases fat metabolism and, therefore, increases the occurrence of obesity (Oh et al., 2010; Ichimura et al., 2012). Hence, germ-free mice possess diminished concentrations of CCK, GLP1 and PYY, which decreases the ability of germ-free mice to sense nutrients within the gut and send regulatory feedback signals through the gut-brain axis and, therefore, leads to enhanced food-intake (Duca et al., 2012; Swartz et al., 2012). Additionally, Fredborg et al. (2012) demonstrated that GPR120 can be upregulated and that GLP1 expression can decrease in the presence of specific bacterial strains *in vitro*. This suggests that changes in microbiota composition may alter intestinal nutrient-sensing and gut-hormone synthesis. While this hypothesis is yet to be established, further studies are needed to validate this and the role of receptors such as GPR120 and the mechanisms that their activation initiates.

Prebiotics, such as oligofructose, improve gut-barrier function, induce weight-loss and reduce food intake, by improving gut nutrient-sensing mechanisms that initiate these effects (Bauer et al., 2016). It has been suggested that these effects are driven by alterations in the gut microbiota composition. Studies have demonstrated diminished levels of specific types of bacteria and gut-derived peptides in the obesogenic state and used this premise in an attempt to restore these bacteria, in addition to increasing the circulating concentrations of gut hormones (Bauer et al., 2016). Prebiotic administration increases *Akkermansia muciniphilia, Faecalibacterium prausnitzii*, Bifidobacterium, and Lactobacilli, which in turn, have been linked to improvements in the gut-barrier function through a glucose-like peptide 2 (GLP2) mediated pathway and an increase in endocannabinoid signalling (Cani et al., 2009; Dewulf et al., 2012). GLP2 is co-secreted with GLP1 from EECs and its release has been positively associated with intestinal growth and function by increasing villus height, crypt-cell depth and proliferation and decreasing enterocyte cell-death (Rowland et al., 2011). Additionally, prebiotic treatment has been associated with improved EEC differentiation and concentrations of GLP1, GIP and PYY, which increase satiety, decrease food consumption, and decrease adiposity (Cani et al., 2005; Neyrinck et al., 2012). Whilst more studies are required to confirm the linkage between manipulation of gut microbiota composition and gut hormone production, there is evidence that the microbiota may influence nutrient-sensing by the GIT, production of gut hormones and stimulation of the gut-brain axis.

The gut microbiota may also communicate to adipose tissue via the endocannabinoid system. Various mouse models have demonstrated that the peripheral endocannabinoid system in the intestinal and adipose tissues, which possesses roles in regulating gut-barrier function and adipogenesis, are regulated by the gut microbiota (Muccioli et al., 2010). Prebiotic administration to select for an increase in Bifidobacterium within obese mice induce a decline in colonic CB1 expression and anandamide concentrations, in addition to increased colonic fatty-acid amide hydrolase (FAAH) expression (Muccioli et al., 2010; Moran and Shanahan, 2014). FAAH is the primary enzyme responsible for the degradation of anandamide. Decreases in these factors within the colon, as well as an increase in FAAH expression, suggests that the gut microbiota may selectively modulate colonic CB1 receptors, which subsequently moderates endocannabinoid tone (Muccioli et al., 2010). More studies are required to replicate these findings and subsequently elucidate how the microbiota may affect the endocannabinoid system and the increased tone associated with the obesogenic state. The increase in these colonic factors may be resultant of enhanced concentrations of gut-derived hormones rather than the direct composition of the microbiota or the microbiota may induce increased signalling of gut hormones to alter changes within the endocannabinoid system via vagal afferent stimulation. Determining the mechanism/s involved may convey additional insights on how the gut-brain axis and the microbiota are linked in relation to energy regulation and therefore can be targeted for the treatment of obesity.

## Lipopolysaccharide, inflammation and microbiota integration into the gut-brain axis

**Figure 4:**
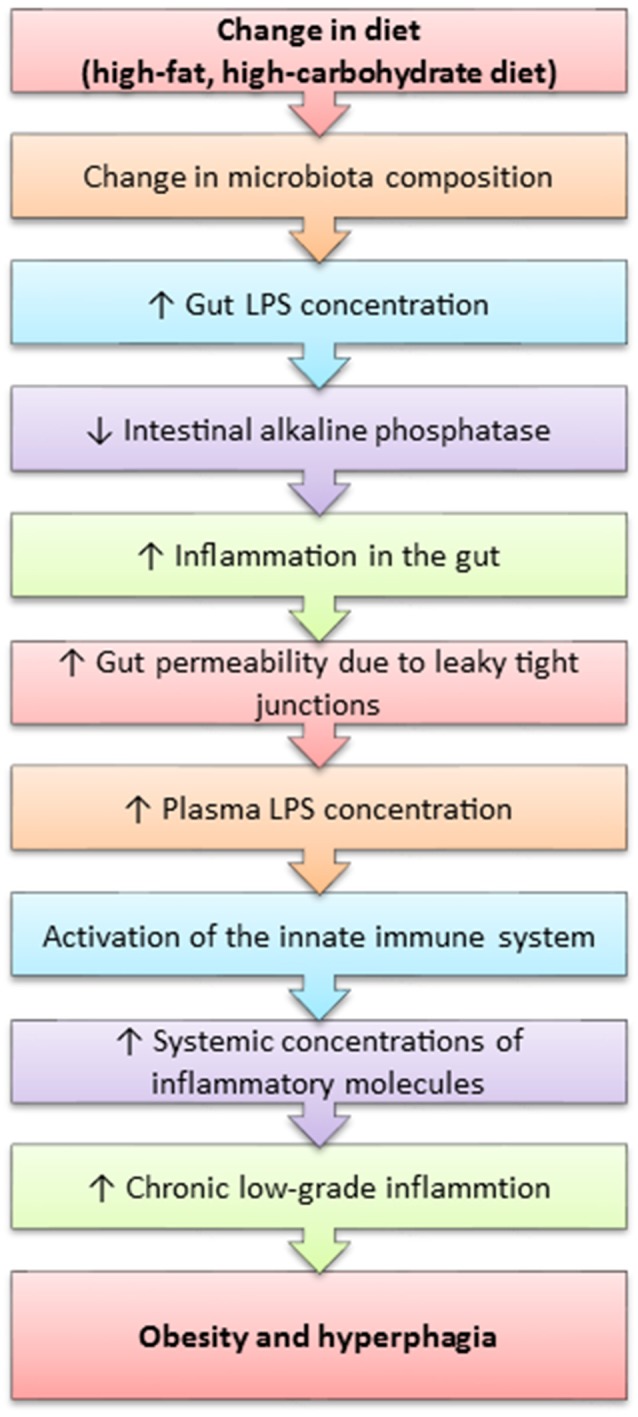
A mechanism outlining how high-fat feeding leads to obesity and polyphagia.

Obesity is considered to be an inflammatory state, as it is characterised by the presence of chronic low-grade inflammation. It has been recently discovered that the westernised-diet, which is high in calories and can lead to obesity and various other obesity-related diseases (Table 1) is associated with elevated systemic lipopolysaccharide (LPS) concentrations (Cani et al., 2007; de La Serre et al., 2010). LPS is the pro-inflammatory component within the cell wall of gram-negative bacteria. LPS is thought to enter the systemic circulation through compromised intestinal epithelial functioning associated with high-fat diets and obesity. This process is termed metabolic endotoxaemia and is hypothesised to occur due to an unfavourable change in the gut microbiota composition, which induces the intestinal epithelium to increase the gap between the junctions formed by each cell, thus increasing gut permeability and permitting the translocation of macronutrients and other molecules, such as LPS (Figure 4) (Cani et al., 2007; de La Serre et al., 2010). The leakage of LPS into the circulation initiates a cascade of pro-inflammatory events throughout the host, especially within white adipose tissue.

LPS is a potent activator of toll-like receptor 4 (TLR4), whose activation results in the synthesis of inflammatory cytokines and subsequent activation of the innate immune system (Vaure and Liu, 2014). Caesar et al. (2015) reported increased systemic LPS concentration, TLR4 activation and white adipose tissue inflammation, as well as reduced insulin-sensitivity and alterations in the gut microbiota composition in mice fed a westernised-diet.

Increased LPS concentrations are concurrent with increased gut concentrations of TLR4 in obese-prone rats (DIO-P), whilst obese-resistant rats (DIO-R) do not express TLR4 within the gut (de La Serre et al., 2010, 2015). The DIO-P rats possessed increases in Clostridiales orders and a decrease in Bifidobacterium, which led to an increase in LPS concentration (de La Serre et al., 2010). de La Serre et al. (2015) reported decreased concentrations of intestinal alkaline phosphatase (IAP) within DIO-P rats, whilst the contrary was observed in the DIO-R rats. This is of significance as IAP is an enzyme native to the gut and responsible for the detoxification of LPS, thus suggesting that LPS may directly act on the gut. This hypothesis is further enhanced by Everard et al. (2014) who demonstrated that intestinal deletion of MyD88, a central adaptor molecule for the majority of toll-like receptors, including TLR4, partially protected obese-prone mice and germ-free mice who received a faecal microbiota transplant from obese-prone mice from diet-induced obesity and inflammation. Additionally, Everard et al. (2014) suggest that deletion of MyD88 may improve nutritional status and provide a therapeutic target for obesity. Further studies are needed to confirm this hypothesis, as TLR4 is also expressed by vagal afferents, thus indicating that LPS may initiate an inflammatory cascade within the neuronal circuitry pertaining to the gut-brain axis (de La Serre et al., 2015).

LPS conveys inhibitory effects on the interstitial cells of Cajal, which function as a pacemaker, creating a slow-wave potential leading to smooth muscle contraction in the gut (peristalsis) and regulation of the ENS (Zuo et al., 2013; Bauer et al., 2016). Inhibition of these cells are associated with gastrointestinal motility disorders, by altering the frequency of neurotransmitter release of neurons within the ENS, which may affect the release of gut hormones and, therefore, link the gut microbiota to impaired gut-brain axis signalling mechanisms (Zuo et al., 2013). Further studies are needed to confirm this hypothesis, as the mechanisms linking this particular region of the peripheral nervous system to the CNS and the gut-brain axis are unknown. Future studies could, for example, explore the option of restoring function to the interstitial cells of Cajal by possibly increasing IAP concentration through intestinal infusion to degrade LPS formation, which may assist in decreasing inflammatory bowel disorders associated with obesity.

**Figure 5:**
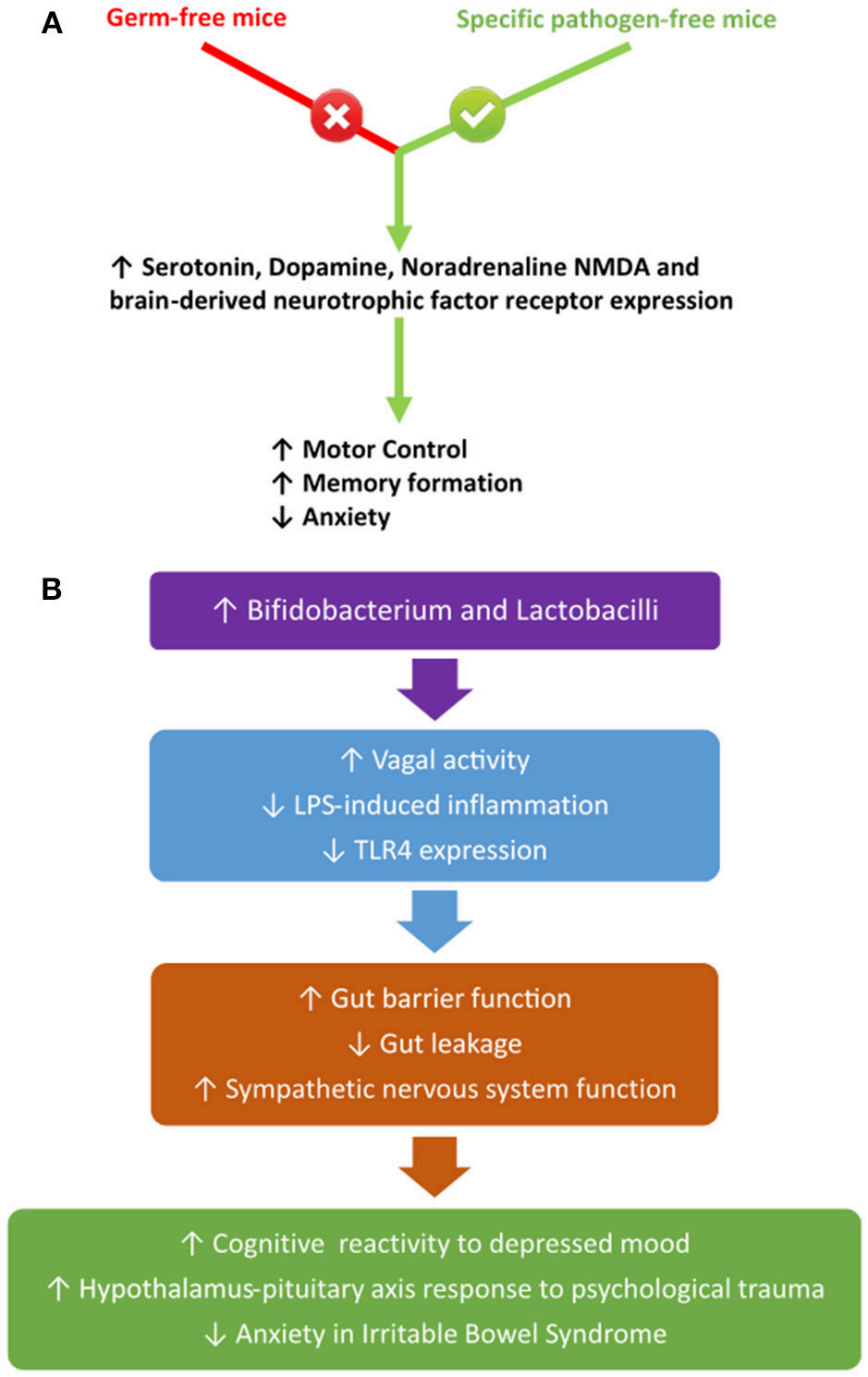
The influence of different bacterial species on the vagus nerve **(A)** and its systemic impact **(B)**.

The gut microbiota may directly communicate and alter CNS signalling mechanisms. Whilst it is still relatively unclear the impact the gut microbiota may have on CNS signalling, with respect to regulation of energy homeostasis, it is becoming evident that the gut microbiota can influence CNS-mediated stress and anxiety behaviours (Bauer et al., 2016). Studies have established differences between germ-free mice and specific pathogen-free mice, which include motor control, memory formation and anxiety due to central neurochemical changes, in particular, those involving brain-derived neurotrophic factor, serotonin, dopamine, noradrenaline, and NMDA receptor expression (Bercik et al., 2010; Heijtz et al., 2011; Neufeld et al., 2011; Steenbergen et al., 2015; Wang and Wang, 2016). Additionally, probiotic treatment, in particular, treatment selective for an increase in Bifidobacterium and Lactobacilli, reduces anxiety in mice with inflammatory bowel disease, enhances cognitive reactivity to depressed mood in humans and reduces depression scores in patients with irritable bowel syndrome, as well as improving hypothalamus-pituitary axis responses to acute psychological trauma (Ait-Belgnaoui et al., 2012; Steenbergen et al., 2015; Pinto-Sanchez et al., 2017). These alterations in behaviour appear to be correlated with vagal activity and diminished LPS-induced inflammation which improves gut barrier function and prevents gut leakage (Bercik et al., 2010). Whilst it can be proposed that specific-pathogenic bacteria, which increase in number during stress and high-fat feeding, release LPS which activates TLR4 on vagal afferents, thus signalling the hypothalamus and other higher-order centres of the brain to integrate and induce an appropriate behaviour, further studies are needed to confirm this hypothesis. Further, *Lactobacillus rhamnosus* CGMCC1.3724 supplementation amplified fat-loss in obese women, in addition to decreasing systemic concentrations of leptin and the relative abundance of Lachnospiraceae, which is a subfamily of the Firmicutes phylum (Sanchez et al., 2014). These findings were replicated in a later study using the same probiotic, in addition to an increase in both satiety and body self-esteem scores, as well as a decrease in both food cravings and depression (Sanchez et al., 2017). Additionally, the vagus nerve can be activated by non-pathogenic bacteria, such as *Lactobacillus lactis*, and this activation enhances sympathetic nervous system activities, whilst subdiaphragmatic vagotomy in the presence of *Lactobacillus lactis* attenuates these effects (Tanida et al., 2005; Forsythe and Kunze, 2013). A summary of these studies can be seen in Figure 5. The mechanism/s elucidating how non-pathogens activate the vagus nerve is yet to be determined and add further complexity into how this gut-brain-microbiota interaction may arise.

**Figure 6:**
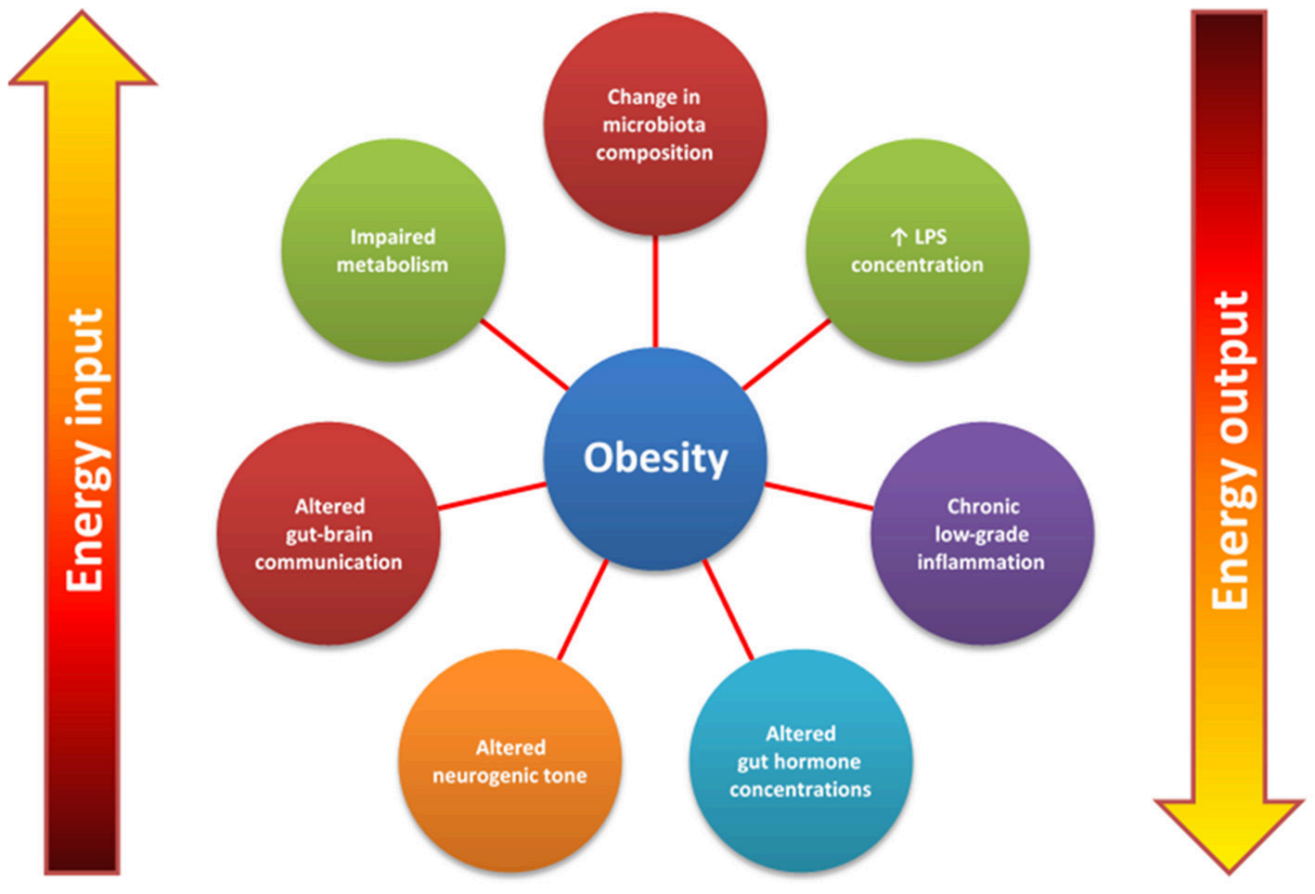
Summary of the effects of an altered microbiota on the gut-brain axis contributing to obesity. This figure summarises the different factors determinants, which have been mentioned throughout this review, that link the gut microbiota with the gut-brain axis in the development of obesity. These include a change in the microbiota composition, increased LPS concentrations culminating in an increase in gut permeability and chronic low-grade inflammation, as well as an increase in energy intake and decrease in energy expenditure.

It is becoming more evident that the gut microbiota impacts CNS functions related to the regulation of energy homeostasis. Bäckhed et al. (2004) demonstrated a resistance to adiposity despite an increased food intake in germ-free mice, thus suggesting that the microbiota directly or indirectly influence the CNS. This finding was the premise for a more elaborate study conducted by Schéle et al. (2013), who compared the gene expression of food intake-regulating peptides and hypothalamic and brainstem feeding circuits between germ-free and normally-reared mice. Schéle et al. (2013) reported a diminished expression of *GCG* which codes for preproglucagon, the precursor peptide for GLP1, GLP2 and oxyntomodulin, within the brainstem and hypothalamus. They also reported diminished leptin-responsiveness in the conventionally-raised mice in comparison to the germ-free mice (Schéle et al., 2013). Additionally, when conventionally-raised mice were treated with leptin, they failed to suppress gene expression of the orexigenic peptides NPY and AgRP within the hypothalamus and brainstem, thus suggesting that the gut-microbiota can directly reduce the expression of anorexigenic peptides and subsequent pathways and affect energy homeostasis, thus leading to an increase in adiposity (Schéle et al., 2013, 2016). Future studies would benefit by determining how specific manipulations of the gut microbiota phenotype can influence the CNS and its role in regulating energy homeostasis and the development of obesity. Additionally, since altered concentrations of gut hormones have been linked to changes in higher neural functions, such as sleep, arousal and anxiety, future studies may be able to link the impact that the gut microbiota conveys on local and central neural-signalling pathways and how this pertains to energy regulation through a microbiota-gut-brain axis (Figure 6) (Forsythe and Kunze, 2013).

## Conclusion

Obesity is a global epidemic that causes socioeconomic strain on governments and public-healthcare systems. Current evidence suggests that life-expectancy will decline as the obesity rate surges and becomes uncontrolled (Olshansky et al., 2005; Grima and Dixon, 2013). Currently, there are limited efficacious treatments available to combat obesity, as current treatment options include alterations in lifestyle and diets, as well as surgical and pharmacological treatments with poorly understood mechanisms resulting in various side-effects (Bauer et al., 2016). Currently, the most effective and sustained treatment option is surgical intervention achieved through gastric bypass surgery, in which the mechanisms leading to its success are poorly understood (Grima and Dixon, 2013). However, analysing alterations in gut hormone concentrations, neuronal circuitry and gut microbiota composition (that is the components of the gut-brain-microbiota axis) post-surgery and why these factors change may assist in the development of new treatment strategies. Given that surgery can increase the population of EECs, which leads to an increased production of peptides and neuronal communication, as well increased post-prandial gut hormone secretion, it is imperative that the dimensions of the gut-brain axis are elucidated to assist in developing future treatments (Mumphrey et al., 2013; Bauer et al., 2016). Additionally, increasing energy expenditure, through the activation of the sympathetic branches of ANS and the possible gut-brain-BAT axis in addition to targeting neurohormone production may be effective in regulating energy balance. Furthermore, studies implicating the role of splanchnic and other somatosensory pathways are needed to be conducted so that it can be understood what role/s these fibres possess within the gut-brain axis, especially given the large presence of these afferents throughout the GIT. However, exploring the exact mechanisms related to this potential axis, ancillary neuronal pathways and fibres, and integrating the pathways involved in relation to the knowledge already obtained regarding the gut-brain axis may prove complex, but with persistence may provide a promising strategy for combating obesity.

Manipulation of the gut microbiota may provide a novel therapeutic strategy in combating obesity and its comorbidities (Bauer et al., 2016). Rapid and persistent shifts in the gut microbiota have been reported to be concomitant with improved metabolic parameters post-surgery (Liou et al., 2013; Osto et al., 2013). Additionally, rodent studies involving the microbiota transplant of post-surgery rodents to germ-free rodents have demonstrated to possess diminished adiposity and increased energy expenditure resulting from modified SCFA production and/or diminished LPS concentrations (Liou et al., 2013; Casselbrant et al., 2015). Whilst manipulating microbiota composition may provide a promising lead in developing anti-obesogenic treatments, more studies are required to elucidate the “ideal” microbiota phenotype with respect to the “healthy” state. Granted prebiotics and probiotics have provided promising insights into the role the microbiota plays within the gut-brain axis and the obesogenic state (Moran and Shanahan, 2014). It is unknown how long the changes in a favourable phenotype may take to occur. Given the “fast-pace” and “convenient” lifestyle associated with modern-living, the compliance associated with taking these supplements may be poor if an improvement is not seen rapidly. Additionally, capsules comprised of what is considered to be an ideal microbiota phenotype, and faecal microbiota transplants have shown promising results in rats and willing participants (Ley et al., 2006; Turnbaugh et al., 2009; Smits et al., 2013). Again, this treatment option may be limited in efficacy, solely due to the psychological aspects that may not have been taken into consideration, such as the simplistic viewpoint that this ultimately is the ingestion of faecal bacteria harvested from a healthy individual. Hence, public education and health promotion programmes would need to be implemented to increase compliance and efficacy. Additionally, these programs could assist in public education with respect to lifestyle choices and the development of the microbiota throughout the lifespan.

Whilst, understanding of the complex interactions associated with the gut-brain-microbiota axis and obesity are in their infancy and understanding of this axis is increasing rapidly, it provides a promising area for future treatments. These advances in knowledge and possible treatment options should complement rather than substitute research addressing the lifestyle and psychological factors that are associated with obesity. This will, therefore, optimistically provide an improved outcome and compliance for future endeavours in alleviating this epidemic.

## Author contributions

EB conceived and wrote the manuscript and EW offered advice and critically revised the manuscript. Both authors approved it for publication.

### Conflict of interest statement

The authors declare that the research was conducted in the absence of any commercial or financial relationships that could be construed as a potential conflict of interest.
